# Maternal Serum tRNA-Derived Fragments (tRFs) as Potential Candidates for Diagnosis of Fetal Congenital Heart Disease

**DOI:** 10.3390/jcdd10020078

**Published:** 2023-02-13

**Authors:** Enkang Lu, Lijun Wu, Bin Chen, Shipeng Xu, Ziyi Fu, Yun Wu, Yanhu Wu, Haitao Gu

**Affiliations:** 1 Department of Cardiothoracic Surgery, The First Affiliated Hospital of Nanjing Medical University, Nanjing 210029, China; 2 Central Laboratory of Jiangsu Provincial Maternal and Child Health Care Hospital, Maternal and Child Branch of the First Affiliated Hospital of Nanjing Medical University, Nanjing 210036, China; 3 Department of Ultrasound, Nanjing Maternity and Child Health Care Hospital, Women’s Hospital of Nanjing Medical University, Nanjing 210004, China; 4 Department of Biomedical Engineering, University of California Davis, Davis, CA 95616, USA

**Keywords:** biomarker, congenital heart disease, tRFs/tiRNAs, cardiac development

## Abstract

Background: Congenital heart disease (CHD) is one of the most predominant birth defects that causes infant death worldwide. The timely and successful surgical treatment of CHD on newborns after delivery requires accurate detection and reliable diagnosis during pregnancy. However, there are no biomarkers that can serve as an early diagnostic factor for CHD patients. tRNA-derived fragments (tRFs) have been reported to play an important role in the occurrence and progression of numerous diseases, but their roles in CHD remains unknown. Methods: High-throughput sequencing was performed on the peripheral blood of pregnant women with an abnormal fetal heart and a normal fetal heart, and 728 differentially expressed tRFs/tiRNAs were identified, among which the top 18 tRFs/tiRNAs were selected as predictive biomarkers of CHD. Then, a quantitative reverse transcriptase polymerase chain reaction verified the expression of tRFs/tiRNAs in more clinical samples, and the correlation between tRFs/tiRNAs abnormalities and CHD was analyzed. Results: tRF-58:74-Gly-GCC-1 and tiRNA-1:35-Leu-CAG-1-M2 may be promising biomarkers. Through further bioinformatics analysis, we predicted that TRF-58:744-GLy-GCC-1 could induce CHD by influencing biological metabolic processes. Conclusions: Our results provide a theoretical basis for the abnormally expressed tRF-58:74-Gly-GCC-1 in maternal peripheral blood as a new potential biomarker for the accurate diagnosis of CHD during pregnancy.

## 1. Introduction

Congenital heart disease (CHD), defined as a general structural abnormality of the heart or large blood vessels present before and/or at birth [1], is the most frequently occurring congenital disorder in newborns and the most common cause of infant death due to birth defects [2,3]. The number of reported births with CHD varies widely by country and region, with the generally accepted best estimate being 6–13 per 1000 live births, and mortality is approximately 40% of prenatal deaths and 20% of deaths in the first year of life [4,5,6,7,8,9,10]. However, the morbidity and mortality of CHD have been greatly reduced with the significant advancement in prenatal and postnatal diagnosis and surgical treatment, such as four-dimensional ultrasonography, fetal cardiac ultrasonography, cardiac MRI, cardiopulmonary bypass technology, etc. However, due to differences in the healthcare system environment, screening professional skills, and available resources, CHD-related examination procedures are often cumbersome, inefficient, and problematic. Moreover, the use of repeat ultrasonography and the application of diagnostic imaging to the potential risk of fetal injury remain controversial [11,12]. Therefore, a new method is urgently needed to ensure the convenience, accuracy, and safety of prenatal screening for a congenital heart defect to exert effective treatment of fetuses born with CHD. A thorough understanding of the molecular mechanism of its occurrence and development could provide tremendous clinical value in both diagnosis and prognosis for CHD patients.

Recently, several promising preliminary studies have identified non-coding RNAs (ncRNAs), including long ncRNAs (lncRNAs) and micro RNAs (miRNAs) that play an important role in cardiac development or the mechanism of CHD occurrence [13,14]. miRNAs are small non-coding RNA molecules that have the potential to regulate 30% of human genes through a complex set of signaling pathways. miRNAs are released into the extracellular circulation via exosomes [15]. Up-regulation of the expression levels of some maternal serum miRNAs has been reported in pregnant women whose fetuses were diagnosed with CHD [16]. As the second most common small ncRNAs after miRNAs, more and more studies have shown that tRFs are active functional molecules. tRFs have stable stem-ring structure, stable riboprotein complexes, and exosome inclusion, and are protected from RNase activity, which allows them to maintain high concentrations in serum, similar to miRNAs [17,18,19,20]. The first tRF found to have a specific biological role in disease was the 3′-tRF, which can be upregulated when affected by HIV [21]. At present, tRFs have been proven to regulate gene expression and promote development, differentiation, inflammation, and tumorigenesis [22,23]. Mature tRNAs or pre-tRNAs are specifically cleaved into tRNA-derived small RNAs (tsRNAs), tRNA-derived fragments (tRFs), and half of tRNAs (tiRNAs) [17]. tRFs are about 14–30 nt in length and contain the 5′-phosphate group and 3′-hydroxyl group, similar to miRNA. tiRNAs are produced by cracking the anticodon ring of the mature tRNAs. Depending on whether the 5′- or 3′-sequence contains anticodon cleavage sites, tiRNAs are divided into 5′-tiRNAs and 3′-tiRNAs subtypes with a length of 31–40 nt [18,19,20]. To date, numerous studies have shown that tRFs/tiRNAs play biological roles through a variety of mechanisms, including the protein or mRNA interactions, regulation of gene expression, control of cell cycle, regulation of chromatin, and epigenetic modification [18]. Similarly, tRFs/tiRNAs also play a crucial role in the occurrence and development of various diseases, such as cancer, skin diseases, and neurodegenerative diseases [24,25,26]. Meanwhile, tRFs/tiRNAs could be considered as potential biomarkers in some diseases, such as breast cancer, multiple myeloma, and dilated cardiomyopathy [27,28,29]. However, the expression profile of tRFs/tiRNAs and its potential role in CHD remain unclear. Therefore, we aimed to assess the probability of tRFs/tiRNAs being diagnostic biomarkers for CHD.

In this study, we investigated the expression profiles of tRFs/tiRNAs in the sera of pregnant females with healthy fetal heart development and pregnant females with CHD-disrupted fetal heart development through RNA-sequencing technology. Subsequently, the differentially expressed tRFs/tiRNAs that met the screening criteria were validated using a quantitative real-time reverse transcription polymerase chain reaction (qRT-PCR). Finally, the potential application value of these fragments in the clinical diagnosis of CHD was analyzed and evaluated.

## 2. Materials and Methods

### 2.1. Clinical Specimen Collection and Processing

The studies involving human participants were reviewed and approved by the Ethics Committee of Women’s Hospital of Nanjing Medical University. All experiments were conducted according to the principles of the Declaration of Helsinki. All patients in this study provided written informed consent. A total of 41 blood samples (N = 41, age = 30.98 ± 3.70) of pregnant women whose fetuses were diagnosed with CHD and 54 blood samples (N = 54, age = 31.62 ± 4.36) of pregnant women with healthy fetal hearts were collected. All CHD diagnoses were verified by fetal echocardiography. Subjects with healthy fetal hearts were recruited from pregnant women participating in prenatal screening. Serum was collected in accordance with Early Detection Research Network operating procedures after obtaining informed consent from patients and healthy individuals. The basic clinical features of the study subjects are shown in Table 1 and Table 2. Whole blood was collected at the outpatient clinic and placed in yellow-capped tubes containing citrate dextrose. It was incubated for 15 min at room temperature and after centrifugation at 3000× *g* for 10 min, serum was transferred to new RNase free tubes. The entire sample processing was performed on ice. Finally, we froze the serum at −80 °C for further experiments [30].

### 2.2. RNA Extraction and Preservation

A fixed volume of serum (200 μL per sample) was used for quantitative reverse transcriptase polymerase chain reaction (qRT-PCR) and tRFs/tiRNAs sequencing detection. Following the manufacturer’s protocol, total RNA was isolated from patient serum with Trizol LS (Life Technologies, Carlsbad, CA, USA) [31]. Then, we confirmed that all OD 260/280 absorbance ratios for NanoDrop ND-1000 were between 1.8 and 2.0. Finally, store the extracted RNA in a −80 °C freezer for subsequent experiments.

### 2.3. Library Preparation and Sequencing

First, the total RNA samples of 6 pregnant women whose fetal diagnosis of CHD (Table 1) and 6 pregnant women with normal fetal heart (Table 2) were preprocessed. In order to achieve efficient reverse transcription, we removed the RNA modifications that interfered with the construction of the small RNA sequence library as follows: 3′-cP (2′,3′-cyclic phosphate) removal to 3′-OH for 3′adaptor ligation; 3′-aminoacyl (charged) diacylation to 3′-OH for 3′ adaptor ligation; 5′-OH (hydroxyl group) phosphorylation to 5′-P for 5′-adaptor ligation and m1A and m3C demethylation. The synthesized cDNA was amplified with RT primers and amplification primers from Illumina. Then, 135–160 bp PCR-amplified fragments (corresponding to the 15–40 nt small RNA size range [32]) were extracted and purified from PAGE gel. Finally, the prepared library was quantified with an Agilent BioAnalyzer 2100 and sequenced with an Illumina NextSeq 500 [33,34].

### 2.4. Bioinformatic Analysis of tRFs/tiRNAs

Image analysis and base calling were performed using Solexa pipeline v1.8 (Off-Line base Caller software, v1.8). Sequencing quality was investigated by FastQC and trimmed reads were aligned to mature tRNA sequences [34]. We filtered the reads for length < 14 nt or length > 40 nt using cutadapt software [35]. The abundance of tRFs/tiRNAs was assessed by sequencing counts and normalized to counts per million aligned reads (CPM). Next, the expression profiles and differential expression of tRFs/tiRNAs were analyzed by R package EdgeR [36], and tRFs/tiRNAs fold change >1.5 and *p* < 0.05 were defined as significant differential expression. R software (v4.1.2) was used to construct heat maps, volcano plots, pie charts, scatter plots, and hierarchical clustering of differentially expressed tRFs/tiRNAs for visual identification [37].

### 2.5. Quantitative Real-Time PCR (qRT-PCR) for tRFs/tiRNA

The sequencing results were verified by qRT-PCR, and the expression levels of tRFs/tiRNAs in serum samples of different pregnant women were determined by the Bulge-loop™ miRNA qRT-PCR Starter Kit (Ribo Bio, Guangzhou, China). The specific experimental method was as follows: First, according to the manufacturer’s instructions, we mixed RT Primer (1 µL), 5X Reverse-Transcription Buffer (2 µL), RNA template (1 µL), RTase Mix (2 µL), and RNase-free H2O (4 µL). Then, the mixture was incubated at 42 °C for 60 min and then at 70 °C for 10 min to obtain the cDNA. Next, cDNA (1 µL) was added to the qPCR along with RNase-free H2O (3.2 µL), Bulge-loop™ miRNA forward primer (0.4 µL), Bulge-loop™ reverse primer (0.4 µL), and 2X SYBR Green mixture (5 µL). The reactions were conducted in the Roche Light Cycler 480 Real Time PCR System (Roche Light Cycler^®^ 480 system, Basel, Switzerland), including incubation at 95 °C for 10 min, followed by 40 cycles of 95 °C for 2 s, 60 °C for 20 s, and 70 °C for 20 s. miDETECT™ miRNA external control (cel-miR-39-3p) was the external control. The 2^−ΔΔCt^ method was used to calculate the expression level of tRFs/tiRNAs in each sample. Details of the tRFs/tiRNAs selected for validation are presented in Table S1.

### 2.6. Receiver Operating Characteristic Analysis

Receiver operating characteristic (ROC) was analyzed using GraphPad Prism 8.0 (GraphPad Software, California, CA, USA) software. The expression level of each selected tRFs/tiRNAs was ranked, showing the patient’s status (1 for CHD, 0 for Healthy). Generate an ROC curve by calculating the area under the curve (AUC) using a binomial exact confidence interval [38].

### 2.7. Functional Analysis of tRFs/tiRNAs

Firstly, to predict the target genes of selected tRFs/tiRNAs, we used the following tools: TargetScan (https://www.targetscan.org/ (accessed on 12 November 2022)), miRanda (http://microrna.org/ (accessed on 12 November 2022)), miRDB (https://mirdb.org/ (accessed on 12 November 2022)) and tRForest (www.Trforest.com (accessed on 12 November 2022)). Then, Gene Ontology (GO) and Kyoto Encyclopedia of Genes and Genomes (KEGG) enrichment analyses were performed using DAVID Bioinformatics Resources (https://david.ncifcrf.gov/ (accessed on 12 November 2022)). Plots were performed using R packages, Finally, we used the String database (https://cn.string-db.org/ (accessed on 12 November 2022)), a database designed for searching interacting genes/proteins, to elucidate the interactions between different genes of interest.

### 2.8. Statistical Analysis

qRT-PCR validation data are presented as mean standard deviation. Data management and analysis were performed using GraphPad Prism 8.0 (GraphPad Software, California, CA, USA). Statistical differences were measured by unpaired Student’s *t*-test. A *p*-value < 0.05 was considered statistically significant. All experiments were repeated three times to ensure the accuracy of the results.

## 3. Results

### 3.1. Catalogue of tRFs/tiRNAs Expressed in Pregnant Women with Fetal Normal Heart and Fetal CHD

**Figure 1:**
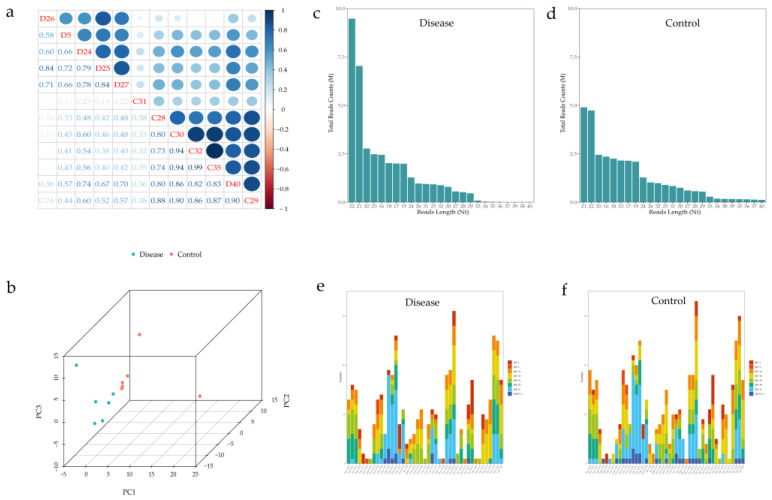
Correlation of samples and catalogue of tRFs/tiRNAs expressed in pregnant women with abnormal fetal heart and normal fetal hearts. (**a**) Coefficient of correlation between samples. (**b**) Principal component analysis (PCA). (**c**,**d**) The read length. (**e**,**f**) Stack diagram of all subtypes of each tRF/tiRNA, clustered by anti-codon of tRNA. The colored bars represent the number of tRFs/tiRNAs per subtype.

High-throughput sequencing technology was used to analyze the expression levels of tRFs/tiRNAs in the disease and control groups. High-throughput sequencing data has been uploaded to the Gene Expression Omnibus (GEO) database, and the accession number is GSE221349 (https://www.ncbi.nlm.nih.gov/geo/query/acc.cgi?acc=GSE221349 (uploaded on 17 December 2022 and acquired accession number on 19 December 2022)). To examine the sequencing quality, the quality (Q) score plot of each sample was plotted (Figure S1). Q30 means the incorrect base calling probability to be 0.001 or 99.9% base calling accuracy. A Q-score > 30 was considered high-quality data. The correlation coefficient of samples is an important evaluation criterion to measure the reliability and reasoning ability of sample selection. The closer the correlation coefficient is to one sample, the more similar the two comparison samples are. Based on the expression level of each sample, the correlation coefficient between any two samples in all samples was calculated (Figure 1a). The results showed that the correlation coefficients within the disease group and within the control group were both high, while the correlation coefficients between the groups were both low, indicating good biological replication of the samples. Principal Component Analysis (PCA) is a statistical method for unsupervised analysis to reduce the dimensionality of large datasets and is a useful tool for exploring sample classes based on expressions. The results of PCA showed that the expression profiles of tRFs/tiRNAs were significantly different between samples (Figure 1b). We used bar graphs to represent the read length distribution for each group sequence (Figure 1c,d). As the tRNA decoder has the same anti-codon, a tRFs/tiRNAs with the same sequence may be the cleavage product of different tRNA genes. 728 tRFs/tiRNAs were identified and mapped to 460 unique tRNAs in the two groups (Table S2). The stacked bar graph is used to compare the number of all tRFs/tiRNAs subtypes with the tRNA decoder, and the frequency of subtypes was compared with the length of tRFs/tiRNAs (Figure 1e,f).

### 3.2. Differentially Expressed tRFs/tiRNAs between Pregnant Women with Fetal Normal Heart and Fetal CHD

**Figure 2:**
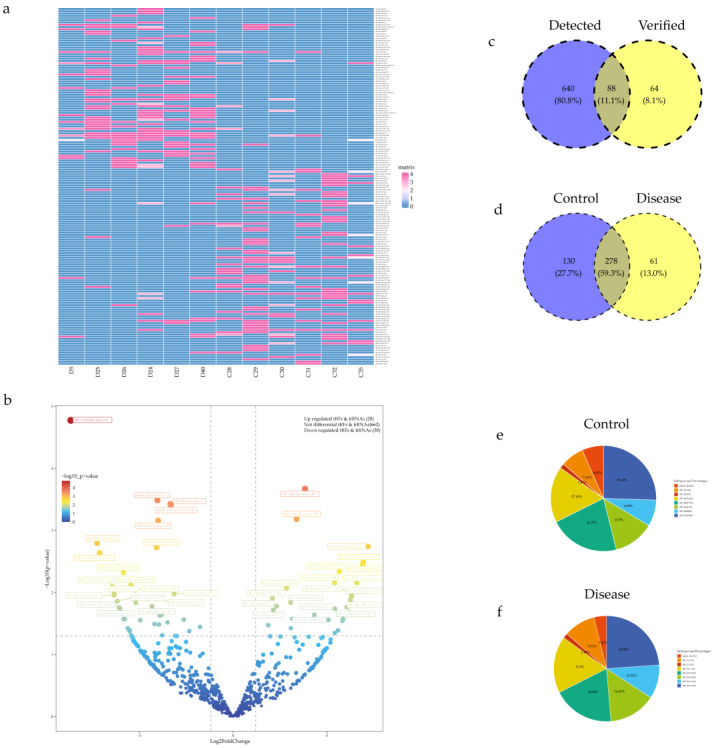
Differential expression of tRFs/tiRNAs between pregnant women with abnormal fetal heart and normal fetal hearts. (**a**) Heatmaps of aberrantly expressed tRFs/tiRNAs in pregnant women with fetal heart defects and normal fetal hearts. (**b**) Volcanic diagrams of aberrantly expressed tRFs/tiRNAs in pregnant women with fetal heart defects and normal fetal hearts, 66 tRFs/tiRNAs were reported to be dysregulated in group disease compared with group control, of which 28 were up-regulated and 38 were down-regulated. (**c**) Out of a total of 728 differentially regulated tRFs/tiRNAs detected, 88 could be found in tRFdb, and the rest were unknown. (**d**) 61 tRFs/tiRNAs specifically expressed in group disease and 130 tRFs/tiRNAs specifically expressed in group control. (**e**,**f**) The percentage of expression of each isoform of tRFs/tiRNAs in the two groups of samples.

In this study, out of a total of 728 differentially regulated tRFs/tiRNAs detected, 88 could be found in tRFdb (http://genome.bioch.virginia.edu/trfdb/ (accessed on 10 September 2022)), and the rest were unknown (Figure 2c). The commonly expressed tRFs/tiRNAs represent the CPM values which were more than 20 in both two groups, and the specifically expressed tRFs/tiRNAs represent the CPM values which were more than 20 in one group while less than 20 in the other group. There were 61 tRFs/tiRNAs specifically expressed in group disease and 130 tRFs/tiRNAs specifically expressed in group control (Figure 2d). The pie chart shows the percentage of expression of each isoform of tRFs/tiRNAs in the two groups of samples; we found that the CHD group mainly increased the expression of tRF-5a, tRF-5b and tRF-1, and decreased the expression of tiRNA-5 and tRF-3b and tRF-5c (Figure 2e,f). Then, we analyzed the characteristics of tRFs/tiRNAs expression by drawing heatmap and volcano plots. A heatmap suggested that there were significant differences in the expression of tRFs/tiRNAs between the two groups (Figure 2a). Under log2FoldChange >1.5 and *p* value < 0.05 criteria, 66 tRFs/tiRNAs were reported to be dysregulated in group disease compared with group control, of which 28 were up-regulated and 38 were down-regulated. The remaining 662 were excluded because they did not meet the criteria (Figure 2b).

**Figure 3:**
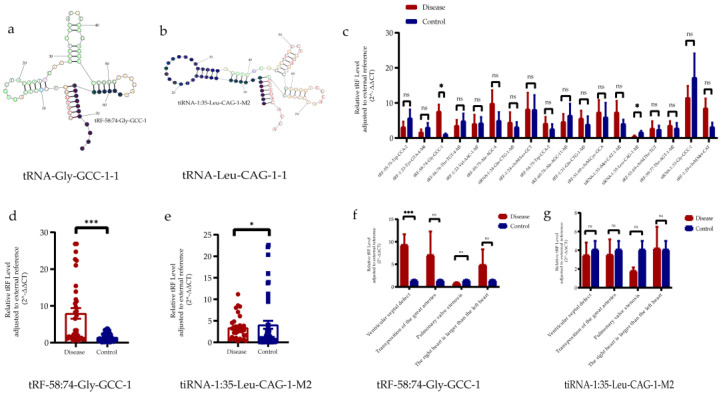
Verification of 2 regulatory anomalies tRFs/tiRNAs in pregnant women with abnormal fetal heart and normal fetal hearts. (**a**,**b**) The position of each tRFs/tiRNAs on the shamrock secondary structure derived from tRNA is shown. tRFs/tiRNAs expression in serum of pregnant women with abnormal fetal heart and normal fetal hearts was analyzed by qRT-PCR. All data were analyzed by Student’s *t* test. The asterisk indicates how significant the difference between the groups is (* *p* < 0.05, *** *p* < 0.001, ns, non-significant). (**c**) Verification of 18 tRFs/tiRNAs (12 Disease vs. 12 Control). (**d**) Verification of tRF-58:74-Gly-GCC-1 (35 Disease vs. 48 Control). (**e**) Verification of tiRNA-1:35-Leu-CAG-1 M2 (35 Disease vs. 48 Control). (**f**,**g**) Verification of tRF-58:74-Gly-GCC-1 and tiRNA-1:35-Leu-CAG-1 M2 for certain CHD defects.

Subsequently, we looked for up- and down-regulated tRFs/tiRNAs in the sequencing results in MINTBASE, and the results showed that five up-regulated tRFs/tiRNAs and 13 down-regulated tRFs/tiRNAs could be looked up in MINTBASE (Table S1). We verified the expression levels of these tRFs/tiRNAs in the peripheral serum of pregnant women (12 disease, 12 control) by qRT-PCR. The qRT-PCR results showed that in the disease group, tRF-58:74-Gly-GCC-1 was up-regulated and tiRNA-1:35-Leu-CAG-1-M2 was down-regulated. We were unable to detect statistically significant differences in the expression of other tRFs/tiRNAs (Figure 3c). Then, we proceeded to expand the sample size (35 disease, 48 control) for further validation of tRF-58:74-Gly-GCC-1 and tiRNA-1:35-Leu-CAG-1-M2. The qRT-PCR results showed that tRF-58:74-Gly-GCC-1 was significantly up-regulated and tiRNA-1:35-Leu-CAG-1 M2 was significantly down-regulated (Figure 3d,e); both tRFs/tiRNAs expression levels were consistent with the sequencing data, and their positions on the secondary structure of tRNA are shown in Figure 3a,b. We subdivided the collected samples into different types of CHD, and then compared them separately with those of the healthy group. Interestingly, the qRT-PCR results showed that tRF-58:74-Gly-GCC-1 was significantly upregulated in the VSD group compared with the control group (Figure 3f,g). However, we did not find tRFs/tiRNAs specific expression in other types of congenital heart disease.

### 3.3. Predictive Value of Specific tRFs/tiRNAs as Diagnostic Biomarkers for CHD

**Figure 4:**
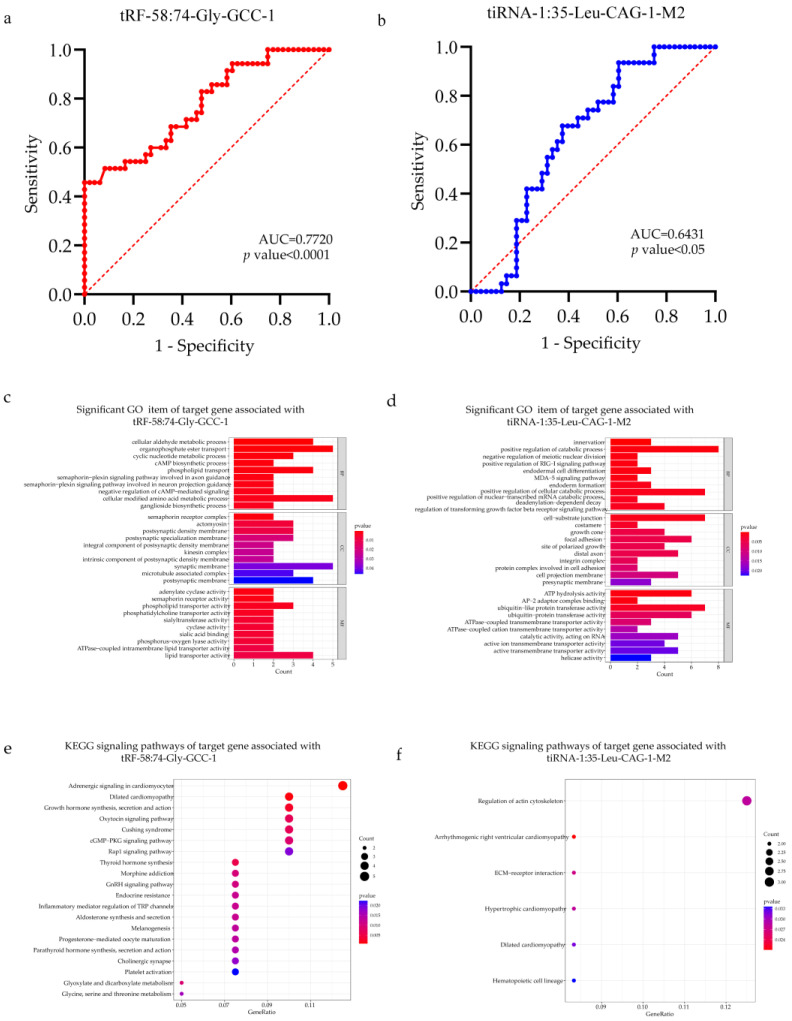
ROC curve, GO and KEGG pathway analysis of specific tRFs/tiRNAs. (**a**,**b**) The vertical axis is sensitivity, the horizontal axis is 1-specificity. AUC is a parameter used to measure the value of tRFs/tiRNAs in the diagnosis of fetal CHD. (**c**–**f**) GO and KEGG pathway analysis of tRF-58:74-Gly-GCC-1 and tiRNA-1:35-Leu-CAG-1-M2.

In order to apply serum tRFs/tiRNAs expression value to clinical diagnosis, ROC curves were analyzed [38]. The ROC curves of patients and healthy women were differentiated according to tRFs/tiRNAs expression levels (Figure 4a,b). AUC value > 0.5 and *p* values < 0.05 can be used as biomarkers [39]. The results show that AUCs of tRF-58:74-Gly-GCC-1 and tiRNA-1:35-Leu-CAG-1-M2 are 0.7720 (*p* value < 0.0001, 95% CI = 0.6703–0.8738) and 0.6431 (*p* value < 0.05, 95% CI = 0.5223–0.7640), respectively. Therefore, we can infer that tRF-58:74-Gly-GCC-1 and tiRNA-1:35-Leu-CAG-1-M2 may be potential candidates for biomarkers of CHD.

### 3.4. Potential Targets and Functional Analysis of Target Genes for Predicting Differential Expression of tRFs/tiRNAs

The potential targets for tRF-58:74-Gly-GCC-1 and tiRNA-1:35-Leu-CAG-1-M2 were identified using the TargetScan, miRanda, miRDB, and tRForest. Then, in order to explore the possible function and mechanism of tRF-58:74-Gly-GCC-1 and tiRNA-1:35-Leu-CAG-1-M2 in the occurrence of CHD, we a performed GO and KEGG pathway analysis of target genes. We classified the target genes of tRFs/tiRNAs based on cellular components, molecular functions, and biological processes. The GO annotation showed that the target genes of tRF-58:74-Gly-GCC-1 were abundant in the organophosphate ester transport and cellular modified amino acid metabolic process (Figure 4c). KEGG annotation showed that the target gene of tRF-58:74-Gly-GCC-1 was highly expressed in adrenergic signaling in cardiomyocytes (Figure 4d). Regarding tiRNA-1:35-Leu-CAG-1-M2, GO annotation indicated that its target gene was highly expressed on positive regulation of catabolic process, positive regulation of cellular catabolic process, cell-substrate junction and ubiquitin-like protein transferase activity (Figure 4e). KEGG annotation showed that the target gene of tRF-58:74-Gly-GCC-1 was highly expressed in the regulation of actin cytoskeleton (Figure 4f). These results may explain their role in fetal heart development and the formation of congenital heart defects.

### 3.5. Protein–Protein Interaction (PPI) Network Analysis

**Figure 5:**
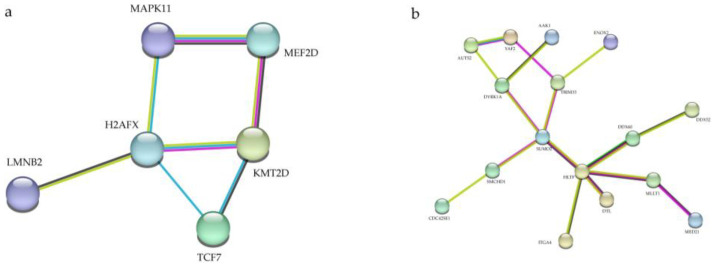
Protein–protein interaction networks of tRF-58:74-Gly-GCC-1 and tiRNA-1:35-Leu-CAG-1-M2. (**a**) The protein–protein interaction network of corresponding target genes oftRF-58:74-Gly-GCC-1 and (**b**) tiRNA-1:35-Leu-CAG-1-M2.

To better understand the underlying mechanisms of tRFs/tiRNAs, we analyzed predicted target gene interactions. First, we analyzed the proteins encoded by 101 target genes of tRF-58:74-Gly-GCC-1 and identified the most frequently interacting proteins H2AFX (Figure 5a). H2AFX, a member of the H2A histone family, is essential for checkpoint mediated cell cycle arrest, DNA repair after DNA double strand breaks, in addition to playing a role in DNA damage repair, DNA fragmentation during apoptosis, and is phosphorylated by different kinases in response to apoptotic signals [40,41,42,43,44]. Thus, we speculate that tRF-58:74-Gly-GCC-1 may play a role in CHD pathogenesis by regulating the activation of the H2AFX pathway. Then, by analyzing 76 genes of tiRNA-1:35-Leu-CAG-1-M2, we found that HLTF was the central protein (Figure 5b). HLTF has chromatin remodeling activity, resulting in the displacement of DNA-binding proteins on stalled replication forks and promoting DNA damage repair. HLTF was specifically expressed in the heart during early embryonic development [45]. Therefore, we speculate that tiRNA-1:35-Leu-CAG-1-M2 and tiRNA-1:35-Leu-CAG-1-M2, may play a role in CHD pathogenesis by regulating the activation of the HLTF pathway.

## 4. Discussion

As the most common congenital defective disorder in newborns, the pathogenesis of CHD is not yet clear [46]. With the continuous improvement of medical conditions and prenatal diagnostic techniques, the vast majority of CHDs can be detected during pregnancy, but it cannot be excluded that there are still some CHDs that cannot be detected throughout pregnancy. In the clinic, some simple types of CHD, such as atrial septal defects, patent ductus arteriosus, and ventricular septal defects, can be treated surgically after birth and have a good prognosis [47]. However, if there is no early detection and treatment of some complex types of CHD, such as hypoplastic left heart syndrome, even after multiple surgeries, the prognosis is poor [48]. Therefore, if we had a serum biomarker for more comprehensive screening and diagnosis of CHD from a different perspective based on routine cardiac ultrasonography, this would largely reduce the prevalence and mortality of CHD. Although many efforts have been made to identify biomarkers for CHD, there are currently no clear biomarkers in the clinic.

With the development of sequencing technology, a new class of ncRNA-tRFs has entered the scene of diagnostic and prognostic biomarkers. tRFs/tiRNAs are an important class of regulatory molecules that play an integral role in cellular functions, such as proliferation, differentiation, apoptosis, and biological processes, such as RNA modification, DNA damage repair, and regulation of gene expression, as well as in the development of many diseases [17,20,49,50,51]. tRFs/tiRNAs are also involved in pathological processes such as viral infection, tumorigenesis, and neurodegeneration. Given that tRFs/tiRNAs are widespread in nature and their expression is closely related to functions such as cell proliferation, tRFs/tiRNAs may be useful biomarkers in some cases. Therefore, we examined the expression of tRFs/tiRNAs in the serum of pregnant women and performed a biological analysis to verify whether tRFs/tiRNAs in the serum of pregnant women could be used as potential biomarkers for CHD.

In this study, we first performed high-throughput sequencing of peripheral serum from pregnant women with fetal heart defects and normal fetal hearts. Based on the sequencing results, we selected the top 18 abnormally expressed tRFs/tiRNAs for qRT-PCR to verify the authenticity of the data. Then, the most up- or down-regulated and statistically significant tRFs/tiRNAs in serum samples from two groups of pregnant women were tRF-58:74-Gly-GCC-1 and tiRNA-1:35-Leu-CAG-1-M2, respectively. The qRT-PCR results showed that tRF-58:74-Gly-GCC-1 was significantly up-regulated in the disease group and tiRNA-1:35-Leu-CAG-1-M2 was down-regulated in the disease group compared to the control group, which was consistent with the sequencing results. Moreover, we unexpectedly found that tRF-58:74-Gly-GCC-1 was specifically expressed in the VSD group. However, abnormal tRFs/tiRNAs expression was not found in other types of CHD in clinical samples. Therefore, we conclude that tRF-58:74-Gly-GCC-1 plays a crucial role in the occurrence and development of ventricular septal defect. The possible reason for our failure to find differentially expressed in tRFs/tiRNAs other types of CHD is the small number of clinical samples. To further understand the underlying mechanism of tRFs/tiRNAs in the development of CHD, we performed GO and KEGG pathway analysis. The results showed that tRF-58:74-Gly-GCC-1 was enriched for target genes in biological processes and adrenergic signaling in cardiomyocytes signaling pathways in cardiomyocytes. Currently, a large body of evidence suggests that CHD occurs as a result of the accumulation of environmental risk factors and an individual’s factors, and that exobiotic metabolic processes such as the elimination of environmental teratogens play an important role in the development and progression of CHD [48,52,53]. Therefore, tRF-58:74-Gly-GCC-1 may be involved in the biological processes leading to the development of CHD. For tiRNA-1:35-Leu-CAG-1-M2, we found that its target genes were mainly enriched in positive regulation of catabolic and positive regulation of cellular catabolic processes, and therefore, tiRNA-1:35-Leu-CAG-1-M2 plays an important role in normal cell growth and development. Since these differential tRFs/tiRNAs biological processes are closely related to the development of CHD, it is particularly important to further explore the relevant molecular mechanisms. Finally, we constructed a PPI network with all target genes predicted by biological analysis in order to further explain the potential mechanisms of tRFs/tiRNAs involvement in CHD development. The results showed that H2AFX and HLTF were the central proteins of tRF-58:74-Gly-GCC-1 and tiRNA-1:35-Leu-CAG-1-M2, respectively. H2AFX plays an important role in DNA damage repair and DNA fragmentation during apoptosis. HLTF also promotes DNA damage repair. Therefore, we conclude that tRF-58:74-Gly-GCC-1 and tiRNA-1:35-Leu-CAG-1-M2 could play a crucial role in DNA damage repair and cell growth and development. In conclusion, our findings suggest that tRF-58:74-Gly-GCC-1 and tiRNA-1:35-Leu-CAG-1-M2 in the peripheral serum of pregnant women are most likely to serve as promising biomarker candidates.

The onset and progress of CHD, mainly in early pregnancy, is a complex and relatively long-term process involving many genetic and epigenetic alterations. To date, there are quite a few types of CHD. Therefore, we need to continue to expand the sample size and collect more detailed pathological features to further identify and analyze different types of CHD. The relationship between the change of tRFs/tiRNAs expression level and the time of pregnancy, the causal relationship between tRFs/tiRNAs expression level and the occurrence of CHD, as well as how the potential downstream targets predicted in this study regulate the biological process also need to be further studied.

## 5. Conclusions

In summary, our study has performed a comprehensive analysis of tRFs/tiRNAs in the sera of pregnant women with fetal heart defects and those with healthy fetal hearts, suggesting that tRF-58:74-Gly-GCC-1, tiRNA-1:35-Leu-CAG-1-M2 may play a significant role in fetal heart development. After further bioinformatics analysis, we could identify tRF-58:74-Gly-GCC-1 as a potential biomarker for CHD. We believe our study could provide insight to further investigate the mechanism of tRFs/tiRNAs in CHD and utilize serum factor for predictive and preventive clinical diagnosis.

## Figures and Tables

**Table 1:** Clinical features for pregnant women with fetal diagnosis of CHD.

Sample Number	Age at Diagnosis	Reproductive History	Gestational Age	Exposure to Harmful Substances	Clinical Diagnosis
**Samples were used for high-throughput sequencing**					
D5	26	G2P1	23W + 3D	NO	Tetralogy of Fallot, Pulmonary atresia
D25	30	G1P0	23W + 2D	NO	Total anomalous pulmonary venous drainage
D26	31	G2P1	23W + 0D	NO	Endocardial cushion defect
D24	35	G1P0	23W + 0D	NO	Tetralogy of Fallot
D27	36	G1P0	24W + 2D	NO	Hypoplastic right heart, Tricuspid Atresia, Pulmonary valve atresia
D40	33	G1P0	24W + 2D	NO	Tricuspid Atresia, Ventricular septal defect
**Samples were used for follow-up qRT-PCR**					
A01	29	G1P0	25W + 5D	NO	Transposition of great arteries, Ventricular septal defect, Aortic valve stenosis with insufficiency of closure
A02	29	G1P0	28W + 0D	NO	Aortic valve stenosis, Right-sided aortic arch
A03	30	G1P0	22W + 6D	NO	Double outlet of the right ventricle
A04	34	G3P1	24W + 4D	NO	Tetralogy of Fallot
A05	29	G1P0	31W + 3D	NO	Ventricular septal defect
A06	27	G1P0	33W + 3D	NO	Pulmonary valve stenosis with incomplete closure
A07	30	G2P1	25W + 1D	NO	Total anomalous pulmonary venous drainage
A08	34	G2P1	26W + 3D	NO	Pulmonary artery crossover
A09	38	G5P1	23W + 6D	NO	Pulmonary valve stenosis
A10	32	G2P1	27W + 3D	NO	Umbilical vein—coronary sinus—right atrium link
A11	36	G2P1	28W + 0D	NO	The right heart is larger than the left heart
A12	23	G1P0	25W + 6D	NO	Ventricular septal defect, Right-sided aortic arch
A13	39	G3P1	33W + 0D	NO	The right heart is larger than the left heart, Aortic arch narrowing
A14	40	G5P4	24W + 3D	NO	Hypoplastic left heart syndrome
A15	29	G1P0	24W + 1D	NO	Pulmonary valve stenosis
A16	34	G2P0	28W + 6D	NO	The right heart is larger than the left heart
A17	29	G2P0	24W + 2D	NO	Moderate tricuspid regurgitation
A18	30	G2P1	23W + 5D	NO	Transposition of the great arteries
A19	29	G1P0	24W + 6D	NO	Ventricular septal defect, Coarctation of the Aorta, Transposition of the great arteries
A20	33	G4P1	25W + 5D	NO	Abnormal development of the aortic arch
A21	31	G1P0	34W + 5D	NO	Hypoplastic right heart
A22	33	G2P1	25W + 3D	NO	The right ventricle is smaller than the left ventricle
A23	29	G1P0	24W + 5D	NO	Aortic arch stenosis
A24	28	G1P0	27W + 4D	NO	Aortic arch stenosis
A25	29	G2P1	28W + 5D	NO	Small left atrium
A26	33	G1P0	25W + 3D	NO	Complete atrial septal defect
A27	33	G2P1	24W + 2D	NO	Ventricular septal defect, Aortic arch stenosis, Possible aortic arch dissection
A28	31	G3P2	27W + 3D	NO	Mid-large tricuspid regurgitation
A29	27	G2P0	22W + 3D	NO	Total anomalous pulmonary venous drainage
A30	27	G1P0	25W + 3D	NO	Ventricular septal defect
A31	24	G1P0	25W + 4D	NO	Transposition of the great arteries, Ventricular septal defect
A32	29	G1P0	24W + 6D	NO	Right-sided aortic arch
A33	31	G1P0	24W + 2D	NO	Ventricular septal defect, Aorta riding across
A34	29	G1P0	28W + 2D	NO	Ventricular septal defect
A35	31	G3P1	24W + 5D	NO	Ventricular septal defect, Aortic valve stenosis

**Table 2:** Clinical features for pregnant women with a healthy fetal heart.

Sample Number	Age at Diagnosis	Reproductive History	Gestational Age	Exposure to Harmful Substances	Whether the Fetal Heart Is Healthy
**Samples were used for high-throughput sequencing**					
C28	34	G2P1	23W + 6D	NO	YES
C29	28	G1P0	24W + 1D	NO	YES
C30	31	G2P0	23W + 6D	NO	YES
C31	28	G1P0	23W + 6D	NO	YES
C32	27	G1P0	24W + 0D	NO	YES
C35	28	G1P0	23W + 6D	NO	YES
**Samples were used for follow-up qRT-PCR**					
B1	34	-	-	NO	YES
B2	35	-	-	NO	YES
B3	34	-	-	NO	YES
B4	36	-	-	NO	YES
B5	33	-	-	NO	YES
B6	28	-	-	NO	YES
B7	26	-	-	NO	YES
B8	36	-	-	NO	YES
B9	34	-	-	NO	YES
B10	36	-	-	NO	YES
B11	32	-	-	NO	YES
B12	33	-	-	NO	YES
B13	48	-	-	NO	YES
B14	30	-	-	NO	YES
B15	31	-	-	NO	YES
B16	31	-	-	NO	YES
B17	31	-	-	NO	YES
B18	28	-	-	NO	YES
B19	36	-	-	NO	YES
B20	36	-	-	NO	YES
B21	35	-	-	NO	YES
B22	31	-	-	NO	YES
B23	18	-	-	NO	YES
B24	34	-	-	NO	YES
B25	30	-	-	NO	YES
B26	36	-	-	NO	YES
B27	36	-	-	NO	YES
B28	32	-	-	NO	YES
B29	27	-	-	NO	YES
B30	28	-	-	NO	YES
B31	31	-	-	NO	YES
B32	31	-	-	NO	YES
B33	32	-	-	NO	YES
B34	33	-	-	NO	YES
B35	32	-	-	NO	YES
B36	41	-	-	NO	YES
B37	30	-	-	NO	YES
B38	30	-	-	NO	YES
B39	30	-	-	NO	YES
B40	31	-	-	NO	YES
B41	34	-	-	NO	YES
B42	32	-	-	NO	YES
B43	27	-	-	NO	YES
B44	30	-	-	NO	YES
B45	33	-	-	NO	YES
B46	26	-	-	NO	YES
B47	27	-	-	NO	YES
B48	27	-	-	NO	YES

## Data Availability

High-throughput sequencing data has been uploaded to the GEO database, and the accession number is GSE221349 (https://www.ncbi.nlm.nih.gov/geo/query/acc.cgi?acc=GSE221349 (uploaded on 17 December 2022 and acquired accession number on 19 December 2022)).

## Supplementary Materials

The following supporting information can be downloaded at: https://www.mdpi.com/article/10.3390/jcdd10020078/s1. Table S1: Details of the tRFs/tiRNAs selected for validation. Table S2: Results of high-throughput sequencing. Figure S1: The quality (Q) score plot of each sample.
