# Supplementary material for: Maternal Serum tRNA-Derived Fragments (tRFs) as Potential Candidates for Diagnosis of Fetal Congenital Heart Disease
Source: J Cardiovasc Dev Dis. 2023 Feb 13;10(2):78. doi: 10.3390/jcdd10020078 (PMC9968204; doi:10.3390/jcdd10020078)
Supplement: Supplementary file 1 [file jcdd-10-00078-s001.zip › jcdd-2141028-supplementary.pdf]

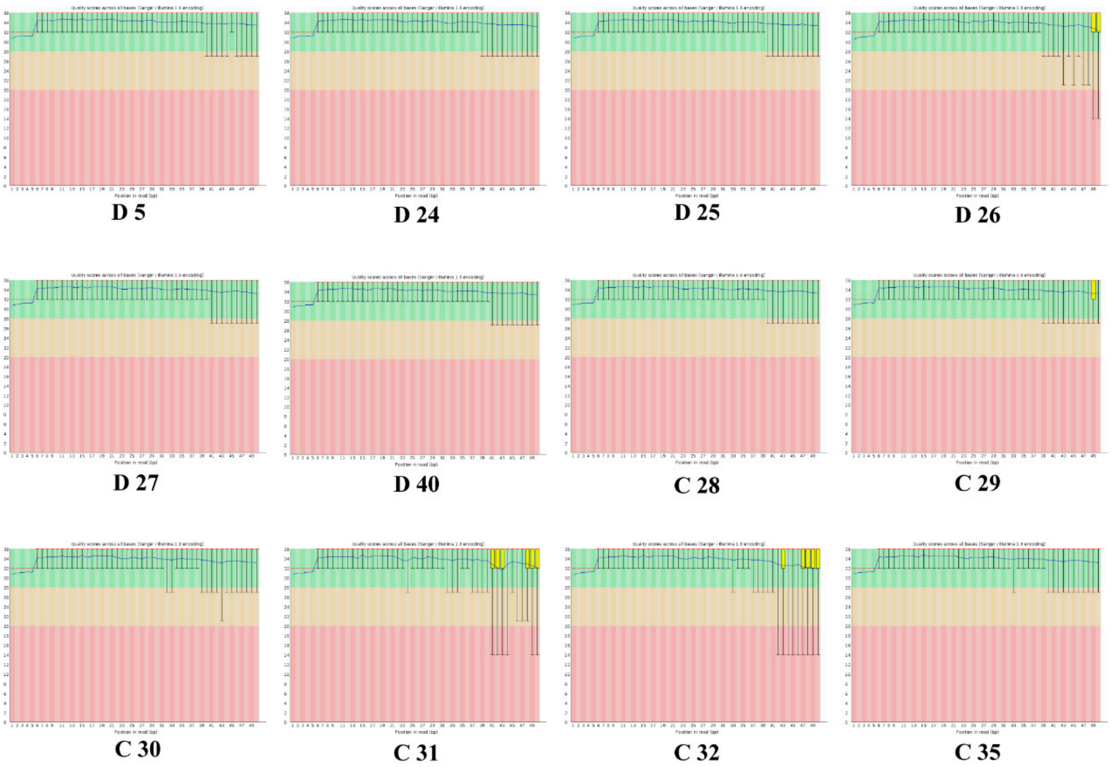


Figure S1. The quality(Q)score plot of each sample. Q30 means the incorrect base calling probability to be 0.001 or 99.9% base calling accuracy. A Q-score > 30 was considered high-quality data.
